# A Case of Abruptio Placentae due to the Torsion of Gravid Uterus

**DOI:** 10.1155/2014/801616

**Published:** 2014-12-04

**Authors:** S. Zullino, S. Faiola, A. M. Paganelli, E. Ferrazzi

**Affiliations:** Vittore Buzzi Children' Hospital, Via Castelvetro 32, 20154 Milan, Italy

## Abstract

Torsion of a gravid uterus is a rare obstetric emergency potentially lethal for the fetus and the mother. Some of the cases described in literature are associated with preexisting gynecologic conditions related to pelvic and uterine anatomy, even if most of cases remain unexplained. We report a case of acute 180-degree torsion of uterus at 33 weeks of gestation associated with abruptio placentae in a young Asian woman without apparent risk factors.

## 1. Introduction

Torsion of gravid uterus is a rare, potentially serious, unexpected, obstetric emergency and it is almost always diagnosed during caesarean section [1]. Uterine torsion is defined as a rotation on its long axis greater than 45 degrees.

When the uterus rotates on its longitudinal axis, the blood supply is dramatically altered due to a vascular obstruction involving at first the veins and after the arteries. This sequence is the cause of acute or subacute abdominal pain, fetal distress, and abruptio placentae in the most severe cases.

There are no reported cohorts of uterine torsions, but only the description of single cases [2–9]. The majority of reported cases of uterine torsion during pregnancy concern patients with uterine leiomyomas [2].

We report a case of acute uterine torsion at 33 weeks of gestation in a singleton pregnancy without uterine myomas.

## 2. Case Presentation

A 25-year-old Asian woman, gravida 2, para 1, reported to our hospital at 33 weeks of gestation with severe abdominal pain. She had a BMI of 24 and a negative obstetric history in the previous pregnancy. The abdominal pain had started three hours prior to admission, like a vague posterior abdominal colicky pain. The patient and her husband did not relate physical efforts prior the onset of the pain. She did not have abdominal surgery in the past. The course of pregnancy was regular including fetal growth.

At the admission in our hospital the patient appeared pale, exhausted, unable to walk, and maintaining an upright position. She had nausea and episodes of vomiting. At triage blood pressure was 90–60 mmHg, heart rate was 120 beats per minute, and temperature was 37.1°C. Abdominal pain was deep, diffuse, with no signs of peritonitis or lateral renal colic. Uterine growth was appropriate for gestational age and the organ was tender on palpation. On vaginal examination cervix was closed and there was no vaginal bleeding or abnormal vaginal discharges.

Ultrasonography showed a single fetus in transverse lie, with a normal amniotic fluid. Placental location was clearly “anterior” with vast subchorionic hypoechogenic area interpreted as a sign of abruptio placentae. Placental tissue was hyperechogenic and thicker than normal. Fetal heart rate at ultrasound examination was 90 beats per minute. It was not possible to visualize and interrogate uterine arteries in their typical anatomical sites by Color Doppler and Pulsed Wave Doppler.

An emergency caesarean section under general anesthesia was performed. Abdomen was opened by standard Stark incision. Peritoneal cavity contained approximately 100 milliliters of coagulated blood. Hysterotomy was done on “the lower uterine segment.” At amniorrhexis the amniotic fluid was clear. A male fetus in transverse lie was delivered by breech extraction. The fetus was alive but pale and hypotonic. A large quantity of fresh clots leaked from the uterus during the afterbirth. The placenta showed a large subchorionic haematoma. The uterus was ischemic and extremely floppy. At exteriorization of the uterus we observed the anterior crossing over of the proper ovarian ligaments. At that time we realized that the uterus was rotated by 180 degrees. We performed the counterclockwise detorsion of the uterus and confirmed a low transverse incision of the posterior wall of the organ. In a matter of seconds after detorsion the myometrium reverted to a normal colour and consistency. We also observed a superficial bleeding laceration of the left part of the posterior wall that probably was the cause of the hemoperitoneum. There were no myomas or uterine malformations. The posterior uterine incision was closed by a standard two-layer suture. The uterus, after the administration of uterotonic, was properly contracted and it was then replaced into the abdomen. The estimated loss of blood was 1100 milliliters. The placenta was sent for pathologic examination that confirmed the clinical diagnosis of abruptio.

Maternal conditions were stable after two hours. Haemoglobin concentration decreased from 10.8 grams/liter at admission to 8.5 grams/liter. White blood cell count was 19.000/milliliter, almost halved from admission values (31.000/milliliter). C-reactive protein had always been negative. Analysing the previous reports and ultrasound images in the patient's pregnancy record, we confirmed that a posterior placental position had been observed at 22 weeks' scan, exactly the opposite of the anterior position we have seen by ultrasound at the time of hospital admission.

The newborn at birth weighted 2200 grams, Apgar score was 1, 3, and 5 at 1, 5, and 10 minutes, cord pH was 6.61, and base deficit was 28 millimol/liter.

A severe acute cerebral haemorrhage was diagnosed. The newborn was admitted at Neonatal Intensive Care Unit (NICU), never recovered his vital functions, and died sixty days after birth.

The woman was discharged three days after caesarian section.

## 3. Discussion

Although the torsion of gravid uterus is a real emergency in obstetrics, it is not easy to study the real epidemiology of this disease due to its rarity. Even if it is not possible to find a clear etiology, the most common risk factors are myomas, uterine malformations, pelvic adhesions, ovarian cysts, abnormal fetal presentation, or anomalies and maternal abnormalities of the spine and the pelvis. Other causes like external cephalic version and maternal trauma have been reported [2, 4]. However the majority of the cases are unexplained.

In our case the only risk factor was the transverse lie of the fetus.

Because of its rarity and the emergency of the situation, it is very difficult to diagnose this condition antenatally. The symptoms reported in literature are persistent abdominal pain associated with a tender uterus on palpation, vaginal bleeding in case placental abruptio is also present, shock, and urinary or intestinal symptoms. Sometimes this condition could be even asymptomatic [9].

The modification of placental site compared to the previous scan on ultrasound and the abnormal position of ovarian and uterine vessels across the uterus on Doppler evaluation can help to suspect the torsion.

The magnetic resonance imaging could be helpful but it is not feasible in emergency situation when fetal distress is suspected [2, 4].

The management of torsion of gravid uterus consists in emergency laparotomy. As described above, it is frequent to diagnose the condition only after the extraction of the fetus. In unexpected cases posterior low transverse incision is usually performed. In such patient with a posterior Hysterotomy an elective caesarean section is advisable for future pregnancy, because the risk of uterine rupture during labour through the posterior scar is not known.

Proposed methods to prevent a recurrence of uterine torsion in future pregnancies, as plication of round ligament or uterosacrals, are not validated [10].

As regards neonatal and maternal complications, Jensen has reported 13% of perinatal mortality [3] and there is one reported case of maternal death in the last fifty years [5].

Even if rare, the severity of this pathology requires knowledge of its existence and awareness of how to handle this emergency. This is the only manner to preserve maternal and newborn's health.

## References

[1] Zanoio L., Eliana B., Gabrio Z. (2007). *Ginecologia e Ostetricia con Tavole di F.H. Netter*.

[2] Deshpande G., Kaul R., Manjuladevi P. (2011). A case of torsion of gravid uterus caused by leiomyoma. *Case Reports in Obstetrics and Gynecology*.

[3] Jensen J. G. (1992). Uterine torsion in pregnancy. *Acta Obstetricia et Gynecologica Scandinavica*.

[4] Wilson D., Mahalingham A., Ross S. (2006). Third trimester uterine torsion: case report. *Journal of Obstetrics and Gynaecology Canada*.

[5] Guié P., Adjobi R., N'Guessan E., Anongba S., Kouakou F., Boua N., Dia J., Kouyaté S., Tegnan J. A., Djanhan L., Bohoussou E., Yao I. (2005). Uterine torsion with maternal death: our experience and literature review. *Clinical and Experimental Obstetrics and Gynecology*.

[6] Simms-Stewart D., Hardie J., Mitchell P., Fletcher H., Reid A., Shah D. (2012). Torsion in a perimenopausal non-gravid uterus with infarction and gangrene of uterus and adnexa: a proposed means of making the diagnosis clinically. *Journal of Obstetrics & Gynaecology*.

[7] Moores K. L., Wood M. G., Foon R. P. (2014). A rare obstetric emergency: acute uterine torsion in a 32-week pregnancy. *BMJ Case Reports*.

[8] Gohil A., Patel M. (2013). Torsion of gravid uterus managed by obstetric hysterectomy with the fetus in situ. *Journal of Obstetrics and Gynecology of India*.

[9] Homam H., Moukhah S., Alizadeh M. (2013). Asymptomatic torsion of a gravid uterus. *Taiwanese Journal of Obstetrics and Gynecology*.

[10] Mustafa M. S., Shakeel F., Sporrong B. (1999). Extreme torsion of the pregnant uterus. *Australian and New Zealand Journal of Obstetrics and Gynaecology*.

